# Epidemiology and Risk Factors of Portal Venous System Thrombosis in Patients With Inflammatory Bowel Disease: A Systematic Review and Meta-Analysis

**DOI:** 10.3389/fmed.2021.744505

**Published:** 2022-01-17

**Authors:** Hanyang Lin, Zhaohui Bai, Fanjun Meng, Yanyan Wu, Li Luo, Akash Shukla, Eric M. Yoshida, Xiaozhong Guo, Xingshun Qi

**Affiliations:** ^1^Department of Gastroenterology, General Hospital of Northern Theater Command (formerly called General Hospital of Shenyang Military Area), Shenyang, China; ^2^China Medical University, Shenyang, China; ^3^Shenyang Pharmaceutical University, Shenyang, China; ^4^Jinzhou Medical University, Jinzhou, China; ^5^Department of Gastroenterology, King Edward Memorial Hospital and Seth Gordhandas Sunderdas Medical College, Mumbai, India; ^6^Division of Gastroenterology, Vancouver General Hospital, Vancouver, BC, Canada

**Keywords:** portal venous system thrombosis, epidemiology, risk factor, inflammatory bowel disease, meta-analysis

## Abstract

**Background:**

Patients with inflammatory bowel disease (IBD) may be at risk of developing portal venous system thrombosis (PVST) with worse outcomes. This study aims to explore the prevalence, incidence, and risk factors of PVST among patients with IBD.

**Methods:**

PubMed, Embase, and Cochrane Library databases were searched. All the eligible studies were divided according to the history of colorectal surgery. Only the prevalence of PVST in patients with IBD was pooled if the history of colorectal surgery was unclear. The incidence of PVST in patients with IBD after colorectal surgery was pooled if the history of colorectal surgery was clear. Prevalence, incidence, and risk factors of PVST were pooled by only a random-effects model. Subgroup analyses were performed in patients undergoing imaging examinations. Odds ratios (ORs) with 95% CIs were calculated.

**Results:**

A total of 36 studies with 143,659 patients with IBD were included. Among the studies where the history of colorectal surgery was unclear, the prevalence of PVST was 0.99, 1.45, and 0.40% in ulcerative colitis (UC), Crohn's disease (CD), and unclassified IBD, respectively. Among the studies where all the patients underwent colorectal surgery, the incidence of PVST was 6.95, 2.55, and 3.95% in UC, CD, and unclassified IBD after colorectal surgery, respectively. Both the prevalence and incidence of PVST became higher in patients with IBD undergoing imaging examinations. Preoperative corticosteroids therapy (OR = 3.112, 95% CI: 1.017–9.525; *p* = 0.047) and urgent surgery (OR = 1.799, 95% CI: 1.079–2.998; *p* = 0.024) are significant risk factors of PVST in patients with IBD after colorectal surgery. The mortality of patients with IBD with PVST after colorectal surgery was 4.31% (34/789).

**Conclusion:**

PVST is not rare, but potentially lethal in patients with IBD after colorectal surgery. More severe IBD, indicated by preoperative corticosteroids and urgent surgery, is associated with a higher risk of PVST after colorectal surgery. Therefore, screening for PVST by imaging examinations and antithrombotic prophylaxis in high-risk patients should be actively considered.

**Systematic Review Registration:**

Registered on PROSPERO, Identifier: CRD42020159579.

## Introduction

Inflammatory bowel disease (IBD) is a chronic and progressive inflammatory disease of the gastrointestinal tract, mainly consisting of ulcerative colitis (UC) and Crohn's disease (CD). In recent years, there has been a rising trend of IBD worldwide, which further increases the economic burden of illness among individual patients, their families, and healthcare systems (1) as well as morbidity and mortality (2). Patients with IBD can experience disease progression from inflammation to stricture or penetration/fistulization (3). Its related complications can also result in poor quality of life (4) and negative emotional impact (5).

Recently, it has been observed that IBD has significant secondary effects on the coagulation cascade, including initiation and propagation of coagulation activation, inhibition of fibrinolysis, and downregulation of physiological anticoagulation pathways (6), which lead to coagulation abnormalities, such as increased levels of coagulation factors V and VIII, platelet count, and fibrin, and a decreased level of antithrombin (7). Patients with IBD have a higher risk of venous thromboembolism (VTE) (8), further increasing their morbidity and mortality (9).

Portal venous system thrombosis (PVST) mainly refers to the development of thrombosis within the portal vein, mesenteric vein, and splenic vein (10). Its clinical manifestations can include complications of acute intestinal ischemia (11), hematemesis or melena from esophagogastric variceal bleeding (12), even multiple organ dysfunction, and death (13). Intraperitoneal inflammation, including pancreatitis (14) and IBD (15), is one of the most common local risk factors for PVST.

It is important for physicians to understand the epidemiology and risk factors of PVST in patients with IBD since such information is potentially helpful to assess and manage this complication in high-risk patients. In this study, this systematic review and meta-analysis aimed to explore the prevalence, incidence, and risk factors of PVST in patients with IBD.

## Methods

This meta-analysis was performed following the Meta-analysis Of Observational Studies in Epidemiology (MOOSE) (16) and the Preferred Reporting Items for Systematic Reviews and Meta-Analyses (PRISMA) (17) statements. The MOOSE and the PRISMA checklists are shown in Supplementary Materials.

### Registration

This study was registered in International prospective register of systematic reviews (PROSPERO) with a registration number of CRD42020159579.

### Search Strategy

All articles concerning PVST in patients with IBD were searched through PubMed, Embase, and Cochrane Library databases. Search terms were as follows: (“portal” or “splenic” or “mesenteric” or “portomesenteric” or “portosplenomesenteric”) and (“vein” or “venous” or “vascular”) and (“thrombosis” or “thrombi” or “thrombus” or “thrombotic” or “thrombosed” or “thromboembolism” or “thromboembolic” or “embolism” or “emboli” or “embolization” or “occluded” or “occlusion” or “occlusive” or “obstructed” or “obstructive” or “obstruction”) and (“inflammatory bowel disease” or “IBD” or “Crohn's disease” or “CD” or “ulcerative colitis” or “UC” or “colitis”). The last retrieval was performed on November 3, 2021.

### Selection Criteria

Eligible studies were included according to the following criteria: (1) patients should be diagnosed with IBD; (2) the number of PVST in patients with IBD can be extracted to calculate the prevalence; (3) the number of PVST after a diagnosis of IBD or colorectal surgery for IBD can be extracted to calculate the incidence; and (4) risk factors associated with the development of PVST in patients with IBD can be extracted. Exclusion criteria were as follows: (1) duplicate article; (2) comment, note, or letter; (3) guideline, consensus, or report; (4) review or meta-analysis; (5) case report; (6) experimental or animal study; (7) full text could not be obtained; (8) patients with IBD were not included; (9) PVST was not evaluated in patients with IBD; (10) relevant data could not be extracted; and (11) overlapping data.

### Definitions

PVST was defined as thrombus occurring in the portal venous system including portal, mesenteric, and splenic vein. The cohort study was defined as the occurrence of PVST events in patients with IBD during follow-up. Cross-sectional study was defined as the presence of PVST events in patients with IBD at a fixed time point. Prevalence of PVST referred to all the PVST conditions in patients with IBD by collecting the data from cohort and cross-sectional studies. Incidence of PVST referred to new onset of PVST events after a diagnosis of IBD or colorectal surgery for IBD by collecting the data from cohort studies.

### Data Extraction

We extracted the following information: first author, publication year, publication type, region, study design, enrollment period, source of case, severity of IBD, history of colorectal surgery, use of antithrombotic drugs, number of patients with IBD, number of patients with IBD who underwent imaging examination, and number of patients with IBD who developed PVST. The characteristics of patients with PVST were further summarized including gender, location of PVST, main clinical presentation, interval from colorectal surgery to diagnosis of PVST, hematological abnormality, treatment selection, and outcome.

### Study Quality

The Newcastle–Ottawa Scale (NOS) was used to evaluate the quality of cohort studies, in which 0–3, 4–6, and 7–9 stars represent low, moderate, and high quality, respectively (18). An 11-item checklist recommended by the Agency for Healthcare Research and Quality (AHRQ) was used to evaluate the quality of cross-sectional studies, in which a score of 0–3, 4–7, and 8–11 represent low, moderate, and high quality, respectively (19).

### Statistical Analysis

All the meta-analyses were conducted by the R software version 3.6.2 (R Core Team, R Foundation for Statistical Computing, Vienna, Austria) and Stata/SE software version 12.0 for Windows (Stata Corporation LP, College Station, Texas, USA). We pooled the prevalence, incidence, and risk factors of PVST in patients with IBD by a random-effects model. Odds ratio (OR) with 95% CI was calculated, if any. *I*^2^ and *p*-value were calculated by inconsistency test to assess the heterogeneity among studies. *I*^2^ > 50% and/or *p* < 0.1 were considered to have a statistically significant heterogeneity. Publication bias was evaluated by Egger's test. *p* < 0.1 was considered as a statistically significant publication bias. Subgroup analyses were performed in terms of region (Europe vs. North America vs. Asia vs. South America vs. Africa), study design (population-based cohort vs. hospital-based cohort vs. cross-sectional study), severity of IBD (exacerbated or refractory), use of antithrombotic drugs, whether the indications of imaging examinations for PVST were mentioned (yes vs. unclear), patients with IBD who underwent imaging examinations, and study quality (high vs. moderate). Meta-regression analyses were performed by the abovementioned covariates to explore the sources of heterogeneity. Sensitivity analyses were also performed by sequentially excluding one study in one turn.

## Results

### Study Selection

**Figure 1:**
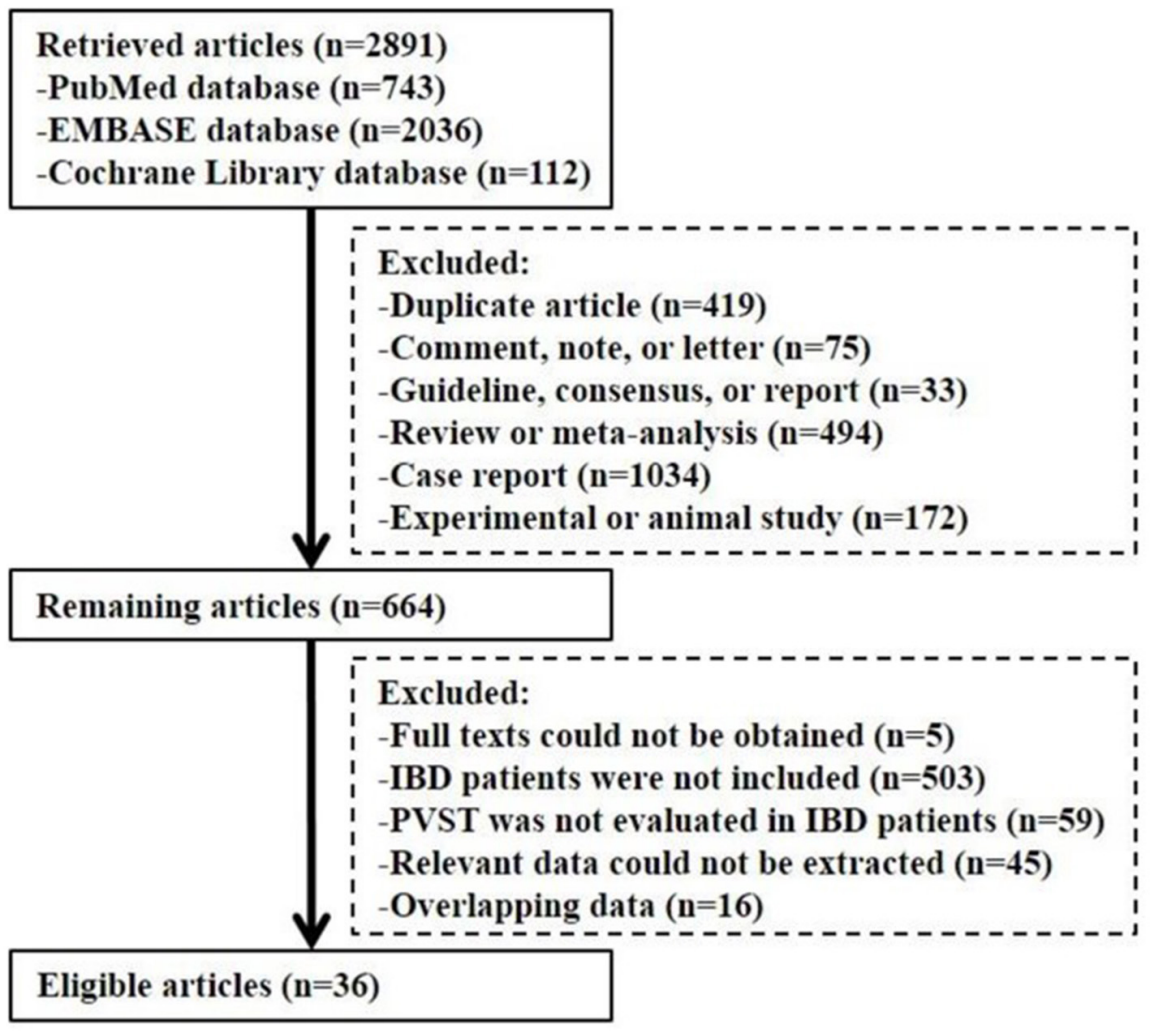
Flowchart of study inclusion.

Overall, 2,891 articles were retrieved. Finally, 36 studies were included (Figure 1). The study quality is given in Supplementary Tables 1, 2.

### Studies Where the Information With Respect to Colorectal Surgery Was Unclear

#### Characteristics of Included Studies

A total of 18 studies where the information with respect to colorectal surgery was unclear can be used to explore the prevalence of PVST in patients with IBD (Table 1) (20–37).

**Table 1:** Characteristics of studies where the information with respect to colorectal surgery was unclear.

**References**	**Publication type**	**Region**	**Study design**	**Enrollment period**	**Source of Pts**.	**No. Pts. PVST/all Pts**.	**Percentage of Pts. With PVST %**	**No. Pts. PVST/Pts. who underwent imaging examinations**	**Study quality**
Ashamalla et al. (20)	Conference abstract	USA	Cross-Sectional	2010– 2014	Two tertiary hospitals of the Northwell health system^a^	132/810 (IBD)	16.30	132/810 (IBD)	Moderate
Banerjee et al. (21)	Conference abstract	India	Cross-Sectional	2004.12–2010.03	Asian Institute of Gastroenterology in Hyderabad	7/569 (UC)	1.23	N/A	Moderate
Blonski et al. (22)	Conference abstract	USA	Cross-Sectional	1997.01–2011.10	Gastroenterology Division for University of Pennsylvania	10/14,674 (IBD)	0.07	N/A	Moderate
Bonnivard et al. (23)	Conference abstract	France	Hospital-Based cohort	2000.01–2012.06	Two French hospitals^a^	1/210 (IBD)	0.48	N/A	High
Bruining et al. (24)	Full text	USA	Hospital-Based cohort	2004.08–2005.10	Miles and Shirley Fiterman Center for Digestive Diseases in Rochester, Minnesota	6/357 (CD)	1.68	6/357 (CD)	Moderate
Campos et al. (25)	Conference abstract	Portugal	Cross-Sectional	2006.08–2013.05	Central Hospital for University of Coimbra	1/774 (IBD)	0.13	N/A	Moderate
Gutta et al. (26)	Conference abstract	USA	Cross-Sectional	2010.01–2014.12	Truman Medical Center for University of Missouri-Kansas City	5/2,408 (IBD)	0.21	N/A	Moderate
Heffley et al. (27)	Conference abstract	UK	Cross-Sectional	2015.04–2015.12	A tertiary care referral center of university^a^	2/84 (IBD)	2.38	N/A	Moderate
Kopylov et al. (28)	Full text	Israel	Hospital-Based cohort	2005– 2010	Chaim Sheba Medical Center	6/460 (CD)	1.30	N/A	Moderate
Leustean et al. (29)	Conference abstract	Romania	Cross-Sectional	2012.06–2017.06	St Spiridon Hospital in Lasi	1/238 (IBD)	0.42	N/A	Moderate
Mouelhi et al. (30)	Conference abstract	Tunisia	Cross-Sectional	2000.01–2015.01	Charles Nicolle Hospital in Tunis	2/295 (IBD)	0.68	N/A	Moderate
Papay et al. (31)	Full text	Austria	Hospital-Based cohort	2006.06–2008.12	Fourteen participating centers in Austria	7/2784 (IBD)	0.25	N/A	High
Ribas Andrade et al. (32)	Conference abstract	Brazil	Hospital-Based cohort	2010.01–2015.10	Clinical Hospital for University of São Paulo	8/781 (CD)	1.02	N/A	Moderate
Sabban et al. (33)	Conference abstract	Argentina	Hospital-Based cohort	1996– 2007	Hospital Italiano in Buenos Aires	1/51 (IBD)	1.96	N/A	High
Soteriadou et al. (34)	Conference abstract	UK	Hospital-Based cohort	2009.06–2012.12	Pennine Acute Hospital NHS Trust in Manchester	2/385 (CD)	0.52	2/385 (CD)	Moderate
Talbot et al. (35)	Full text	USA	Hospital-Based cohort	1970.01–1980.12	Mayo Clinic in Rochester, Minnesota	8/7,199 (IBD)	0.11	N/A	Moderate
Vegh et al. (36)	Full text	Hungary	Population-Based cohort	1977.01–2012.12	Five general hospitals and gastroenterology outpatient units in Veszprem province	1/1,060 (UC) 1/648 (CD)	0.09 0.15	N/A	High
Violi et al. (37)	Full text	Switzerland	Hospital-Based cohort	2006.07–2011.06	Swiss IBD Study Cohort at Lausanne University Hospital	8/39 (UC) 35/121 (CD)	20.51 28.93	8/39 (UC) 35/121 (CD)	High

a *Detailed information of hospitals cannot be found*.

*PVST, portal venous system thrombosis; Pts, patients; USA, United States of America; N/A, not applicable; UC, ulcerative colitis; CD, Crohn's disease; IBD, inflammatory bowel disease; UK, United Kingdom*.

#### Characteristics of Patients With PVST

Overall, 244 of 33,947 patients with IBD had PVST (20–37). Among them, PVST was located at the main portal vein and mesenteric vein and its branches in 106 (43.44%) and 147 (60.25%) patients, respectively; bowel stenosis, perianal fistula, internal fistula, and perianal abscess were observed in 24 (9.84%), ten (4.10%), seven (2.87%), and seven (2.87%) patients, respectively, and three (1.23%) patients died during follow-up (Supplementary Table 3).

#### Ulcerative Colitis

A total of three studies evaluated patients with UC (21, 36, 37). The pooled prevalence of PVST in patients with UC was 0.99% (Table 2). One study reported a detailed number of patients who underwent imaging examinations for PVST (37), with a prevalence of 20.51%. Meta-regression and sensitivity analyses were not performed due to a small number of included studies.

**Table 2:** Prevalence of PVST in patients with IBD in whom the information with respect to colorectal surgery was unclear.

**Groups**	**No. studies**	**Range**	**Pooled proportion using random-effects model**	**Heterogeneity**		**Publication bias Egger test (*P*-value)**
				***I* ^2^**	***P*-value**	
**UC**	3	0.0009–0.2051	0.0099 (95% CI 0–0.0274)	87.30%	0.0004	0.0115
**Region (Europe vs. Asia)**						
Europe	2	0.0009–0.2051	0.0928 (95% CI 0–0.2919)	90.00%	0.0016	N/A
Asia	1	N/A	0.0123 (95% CI 0.0050–0.0252)	N/A	N/A	N/A
**Study design (population-based cohort vs. hospital-based cohort vs. cross-sectional)**						
Population-Based cohort	1	N/A	0.0009 (95% CI 0–0.0052)	N/A	N/A	N/A
Hospital-Based cohort	1	N/A	0.2051 (95% CI 0.0930–0.3646)	N/A	N/A	N/A
Cross-Sectional	1	N/A	0.0123 (95% CI 0.0050–0.0252)	N/A	N/A	N/A
**Whether the detailed number of patients who underwent imaging examinations was reported (Yes)**						
Yes	1	N/A	0.2051^a^ (95% CI 0.0930–0.3646)	N/A	N/A	N/A
**Study quality (high vs. moderate)**						
High	2	0.0009–0.2051	0.0928 (95% CI 0–0.2919)	90.00%	0.0016	N/A
Moderate	1	N/A	0.0123 (95% CI 0.0050–0.0252)	N/A	N/A	N/A
**CD**	6	0.0015–0.2893	0.0145 (95% CI 0.0026–0.0263)	91.60%	<0.0001	0.0136
**Region (Europe vs. North America vs. Asia vs. South America)**						
Europe	3	0.0015–0.2893	0.0273 (95% CI 0.0015–0.0531)	95.90%	<0.0001	0.2412
North America	1	N/A	0.0168 (95% CI 0.0062–0.0362)	N/A	N/A	N/A
Asia	1	N/A	0.0130 (95% CI 0.0048–0.0282)	N/A	N/A	N/A
South America	1	N/A	0.0102 (95% CI 0.0044–0.0201)	N/A	N/A	N/A
**Study design (population-based cohort vs. hospital-based cohort)**						
Population-Based cohort	1	N/A	0.0015 (95% CI 0–0.0086)	N/A	N/A	N/A
Hospital-Based cohort	5	0.0052–0.2893	0.0222 (95% CI 0.0047–0.0397)	91.80%	<0.0001	0.0145
**Whether the detailed number of patients who underwent imaging examinations was reported (Yes)**						
Yes	3	0.0052–0.2893	0.0623 (95% CI 0.0158–0.1088)	95.90%	<0.0001	0.1340
**Study quality (high vs. moderate)**						
High	2	0.0015–0.2893	0.1425 (95% CI 0–0.4243)	97.90%	<0.0001	N/A
Moderate	4	0.0052–0.0168	0.0096 (95% CI 0.0053–0.0139)	0.80%	0.3880	0.1904
**Unclassified IBD**	11	0.0007–0.1630	0.0040 (95% CI 0.0014–0.0067)	94.00%	<0.0001	0.0651
**Region (Europe vs. North America vs. South America vs. Africa)**						
Europe	5	0.0013–0.0238	0.0023 (95% CI 0.0008–0.0037)	0%	0.5911	0.1334
North America	4	0.0007–0.1630	0.0052 (95% CI 0.0011–0.0093)	98.10%	<0.0001	0.1531
South America	1	N/A	0.0196 (95% CI 0.0005–0.1045)	N/A	N/A	N/A
Africa	1	N/A	0.0068 (95% CI 0.0008–0.0243)	N/A	N/A	N/A
**Study design (hospital-based cohort vs. cross-sectional)**						
Hospital-Based cohort	4	0.0011–0.0196	0.0015 (95% CI 0.0005–0.0024)	8.40%	0.3512	0.1096
Cross-Sectional	7	0.0007–0.1630	0.0105 (95% CI 0.0044–0.0165)	96.30%	<0.0001	0.1525
**Severity of IBD (exacerbation)**						
Exacerbation	1	N/A	0.1630 (95% CI 0.1382–0.1902)	N/A	N/A	N/A
**Whether the detailed number of patients who underwent imaging examinations was reported (Yes)**						
Yes	1	N/A	0.1630^a^ (95% CI 0.1382–0.1902)	N/A	N/A	N/A
**Study quality (high vs. moderate)**						
High	3	0.0025–0.0196	0.0026 (95% CI 0.0008–0.0045)	0%	0.6124	0.1316
Moderate	8	0.0007–0.1630	0.0045 (95% CI 0.0013–0.0076)	95.70%	<0.0001	0.1132

a *Pooled prevalence of PVST in patients who underwent imaging examinations*.

*PVST, portal venous system thrombosis; IBD, inflammatory bowel disease; UC, ulcerative colitis; N/A, not applicable; CD, Crohn's disease*.

#### Crohn's Disease

A total of six studies evaluated patients with CD (24, 28, 32, 34, 36, 37). The pooled prevalence of PVST in patients with CD was 1.45% (Table 2). Three studies reported a detailed number of patients who underwent imaging examinations for PVST (24, 34, 37), with a pooled prevalence of 6.23%. Meta-regression (Supplementary Table 4) and sensitivity analyses (Supplementary Table 5) did not identify any source of heterogeneity.

#### Unclassified IBD

A total of 11 studies evaluated patients with unclassified IBD (20, 22, 23, 25–27, 29–31, 33, 35). The pooled prevalence of PVST in patients with unclassified IBD was 0.40% (Table 2). One study reported a detailed number of patients who underwent imaging examinations for PVST (20), with a prevalence of 16.30%. Meta-regression analyses indicated that the severity of IBD (*p* < 0.001) and whether the detailed number of patients undergoing imaging examinations was reported (*p* < 0.001) might be potential sources of heterogeneity (Supplementary Table 4). Sensitivity analyses found that the heterogeneity became non-significant after excluding the study by Ashamalla et al. (20) (*I*^2^ = 21.80%; *p* = 0.2423; Supplementary Table 5).

#### Risk Factors of PVST in Patients With IBD

A total of two studies demonstrated that disease duration and colorectal surgery might be significant risk factors of PVST (22), but not age, sex, body mass index (BMI), IBD location, corticosteroids therapy, smoking, or family history of IBD (37).

### Studies Where the Information With Respect to Colorectal Surgery Was Clear

#### Characteristics of Included Studies

A total of 18 studies where the information with respect to colorectal surgery was clear were used to explore the incidence of PVST in patients with IBD after colorectal surgery (Table 3) (38–55).

**Table 3:** Characteristics of studies where all the included patients underwent colorectal surgery.

**References**	**Publication type**	**Region**	**Study design**	**Enrollment period**	**Source of Pts**.	**No. Pts. PVST/all Pts**.	**Percentage of Pts. With PVST %**	**No. Pts. PVST/ Pts. who underwent imaging examinations**	**Study quality**
Allaix et al. (38)	Full text	USA	Hospital-Based cohort	2002.06–2012.06	Surgery Department for University of Chicago Medical Center in Illinois	23/447 (UC) 11/304 (CD)	5.15 3.62	N/A	High
Ball et al. (39)	Full text	Canada	Hospital-Based cohort	1997.01–2002.12	Foothills Medical Center for University of Calgary in Alberta	11/112 (UC)	9.82	11/28 (UC)	Moderate
Bence et al. (40)	Full text	USA	Hospital-Based cohort	2010.01–2016.06	Four tertiary care children's hospitals	9/276 (IBD)	3.26	N/A	High
Feuerstein et al. (41)	Conference abstract	USA	Hospital-Based cohort	2002.01–2014.01	Beth Israel Deaconess Medical Center in Boston, Massachusetts	8/259 (UC)	3.09	N/A	High
Fichera et al. (42)	Full text	USA	Hospital-Based cohort	1999.01–2001.12	Mount Sinai Hospital in New York	4/83 (IBD)	4.82	4/14 (IBD)	Moderate
Gonzales et al. (43)	Conference abstract	USA	Hospital-Based cohort	2003.06–2008.01	Department of Surgery for Boston University School of Medicine in Massachusetts	20/85 (UC)	23.53	20/33 (UC)	Moderate
Gu et al. (44)	Full text	USA	Hospital-Based cohort	2006– 2012	Department of Colorectal Surgery for Cleveland Clinic in Ohio	36/521 (IBD)	6.91	36/216 (IBD)	High
Kayal et al. (45)	Full text	USA	Hospital-Based cohort	2010.01–2016.12	Mount Sinai Hospital in New York	36/434 (UC)	8.29	36/205 (UC)	High
Mathis et al. (46)	Conference abstract	USA	Hospital-Based cohort	2007.09–2012.08	Mayo Clinic in Rochester, Minnesota	4/63 (IBD)	6.35	N/A	Moderate
Mathis et al. (47)	Full text	USA	Hospital-Based cohort	1994– 2005	Mayo Clinic in Rochester, Minnesota	3/100 (IBD)	3.00	N/A	High
Medress and Fleshner (48)	Full text	USA	Hospital-Based cohort	2001.08–2006.08	Cedars-Sinai Medical Center in Los Angeles, California	5/202 (IBD)	2.48	N/A	Moderate
Murphy et al. (49)	Conference abstract	USA	Hospital-Based cohort	2008.01–2012.07	Two clinic institution in Boston, Massachusetts^a^	26/1,014 (IBD)	2.56	N/A	Moderate
Naik et al. (50)	Conference abstract	USA	Hospital-Based cohort	2008– 2010	Gastroenterology and Hepatology Division for Medical College of Wisconsin in Milwaukee	7/131 (IBD)	5.34	N/A	Moderate
Robinson et al. (51)	Full text	USA	Hospital-Based cohort	2007.01–2012.12	Mayo Clinic in Phoenix, Arizona	11/125(UC) 1/78 (CD)	8.80 1.28	N/A	High
Syed et al. (52)	Full text	USA	Population based cohort	1999– 2020.04	Explorys database of IBM, New York	570/105,410 (UC)	0.54	N/A	High
Vaidya et al. (53)	Conference abstract	USA	Hospital-Based cohort	2018.06–2019.07	Department of Colorectal Surgery for Cleveland Clinic in Ohio	1/18 (UC)	5.56	N/A	Moderate
Weisshof et al. (54)	Full text	USA	Hospital-Based cohort	2010.01–2018.03	IBD Center for University of Chicago in Illinois	2/24 (IBD)	8.33	N/A	Moderate
Zaghiyan et al. (55)	Full text	USA	Hospital-Based cohort	2010.01–2010.08	Cedars-Sinai Medical Center in Los Angeles, California	1/26 (IBD)	3.85	N/A	Moderate

a *Detailed information of hospitals cannot be found*.

*Pts, patients; PVST, portal venous system thrombosis; USA, United States of America; UC, ulcerative colitis; CD, Crohn's disease; N/A, not applicable; IBD, inflammatory bowel disease*.

#### Characteristics of Patients With PVST

Overall, 789 of 109,712 patients with IBD developed PVST after colorectal surgery (38–55). Among them, PVST was located at the main portal vein and mesenteric vein and its branches in 20 (2.53%) and 38 (4.82%) patients, respectively; abdominal pain, prolonged ileus, wound infection, and dehydration/sodium depletion was observed in 46 (5.83%), 22 (2.79%), 11 (1.39%), and eight (1.01%) patients, respectively. The interval from surgery to diagnosis of PVST was within 30 days and over 30 days in 45 (5.70%) and two (0.25%) patients, respectively; and 34 (4.31%) patients died during follow-up (Supplementary Table 6).

#### Ulcerative Colitis After Colorectal Surgery

A total of eight studies evaluated patients with UC (38, 39, 41, 43, 45, 51–53). The pooled incidence of PVST in patients with UC after colorectal surgery was 6.95% (Table 4). Three studies reported on the number of patients who underwent imaging examinations for PVST (39, 43, 45) in detail, with a pooled incidence of 38.33%. Meta-regression analyses indicated that whether the detailed number of patients undergoing imaging examinations was reported (*p* = 0.043) might be a potential source of heterogeneity (Supplementary Table 7). Sensitivity analyses did not identify any source of heterogeneity (Supplementary Table 8).

**Table 4:** Incidence of PVST in patients with IBD after colorectal surgery.

**Groups**	**No. studies**	**Range**	**Pooled proportion using random-effects model**	**Heterogeneity**		**Publication bias** **Egger test (*P*-value)**
				***I^**2**^***	***P*-value**	
**UC**	8	0.0054– 0.2353	0.0695 (95% CI 0.0355–0.1036)	93.40%	<0.0001	0.0013
**Region (USA vs. Canada)**						
USA	7	0.0054– 0.2353	0.0654 (95% CI 0.0300–0.1007)	93.70%	<0.0001	0.0045
Canada	1	N/A	0.0982 (95% CI 0.0501–0.1689)	N/A	N/A	N/A
**Study design (population-based cohort vs. hospital-based cohort)**						
Population-Based cohort	1	N/A	0.0054 (95% CI 0.0050–0.0059)	N/A	N/A	N/A
Hospital-Based cohort	7	0.0309– 0.2353	0.0789 (95% CI 0.0480–0.1099)	79.00%	<0.0001	0.0899
**Severity of UC (refractory)**						
Refractory	1	N/A	0.0829 (95% CI 0.0588–0.1130)	N/A	N/A	N/A
**Use of antithrombotic drugs (yes)**						
Yes	1	N/A	0.0841^a^ (95% CI 0.0596–0.1145)	N/A	N/A	N/A
**Whether the indications of imaging examinations for PVST were mentioned (yes vs. unclear)**						
Yes	6	0.0309– 0.2353	0.0810 (95% CI 0.0482–0.1137)	82.50%	<0.0001	0.0348
Unclear	2	0.0054– 0.0556	0.0054 (95% CI 0.0050–0.0059)	0%	0.3530	N/A
**Whether the detailed number of patients who underwent imaging examinations was reported (yes)**						
Yes	3	0.1756– 0.6061	0.3833^b^ (95% CI 0.1058–0.6609)	92.50%	<0.0001	0.2530
**Study quality (high vs. moderate)**						
High	5	0.0054– 0.0880	0.0486 (95% CI 0.0147–0.0825)	94.30%	<0.0001	0.0134
Moderate	3	0.0556– 0.2353	0.1298 (95% CI 0.0330–0.2266)	75.90%	0.0158	0.8334
**CD**	2	0.0128– 0.0362	0.0255 (95% CI 0.0027–0.0483)	49.30%	0.1604	N/A
**Unclassified IBD**	10	0.0248– 0.0833	0.0395 (95% CI 0.0269–0.0521)	45.90%	0.0548	0.1038
**Severity of IBD (refractory)**						
Refractory	4	0.0248– 0.0833	0.0523 (95% CI 0.0206–0.0839)	65.80%	0.0325	0.7107
**Use of antithrombotic drugs (yes)**						
Yes	2	0.0534– 0.0691	0.0653^a^ (95% CI 0.0463–0.0843)	0%	0.4878	N/A
**Whether the indications of imaging examinations for PVST were mentioned (yes vs. unclear)**						
Yes	3	0.0482– 0.0691	0.0628 (95% CI 0.0453–0.0804)	0%	0.6267	0.0324
Unclear	7	0.0248– 0.0833	0.0278 (95% CI 0.0200–0.0356)	0%	0.8232	0.0222
**Whether the detailed number of patients who underwent imaging examinations was reported (yes)**						
Yes	2	0.1667– 0.2857	0.1717^b^ (95% CI 0.1231–0.2203)	0%	0.3346	N/A
**Study quality (high vs. moderate)**						
High	3	0.0300– 0.0691	0.0451 (95% CI 0.0188–0.0714)	70.40%	0.0341	0.7808
Moderate	7	0.0248– 0.0833	0.0288 (95% CI 0.0204–0.0371)	0%	0.5349	0.0120

a *Pooled incidence of PVST in patients who received antithrombotic drugs*.

b *Pooled incidence of PVST in patients who underwent imaging examinations*.

*PVST, portal venous system thrombosis; IBD, inflammatory bowel disease; UC, ulcerative colitis; USA, United States of America; N/A, not applicable; CD, Crohn's disease*.

#### Crohn's Disease After Colorectal Surgery

A total of two studies evaluated patients with CD (38, 51). The pooled incidence of PVST in patients with CD after colorectal surgery was 2.55% (Table 4). Neither study reported on the number of patients who underwent imaging examinations for PVST in detail. Subgroup, meta-regression, and sensitivity analyses were not performed due to a small number of included studies.

#### Unclassified IBD After Colorectal Surgery

A total of ten studies evaluated patients with unclassified IBD (40, 42, 44, 46–50, 54, 55). The pooled incidence of PVST in patients with unclassified IBD after colorectal surgery was 3.95% (Table 4). Two studies reported the detailed number of patients who underwent imaging examinations for PVST (42, 44), with a pooled incidence of 17.17%. Meta-regression analyses indicated that use of antithrombotic drugs (*p* = 0.008), whether the indications of imaging examinations for PVST were mentioned (*p* = 0.007), and whether the detailed number of patients undergoing imaging examinations was reported (*p* = 0.010) might be potential sources of heterogeneity (Supplementary Table 7). Sensitivity analyses found that the heterogeneity became nonsignificant after excluding the study by Gu et al. (44) (*I*^2^ = 0%; *p* = 0.7378) or Murphy et al. (49) (*I*^2^ = 28.10%; *p* = 0.1946) (Supplementary Table 8).

#### Comparison of Incidence of PVST After Colorectal Surgery for IBD and Non-IBD Diseases

A total of two studies included patients who underwent colorectal surgery for IBD, cancer, diverticulitis, and polyps (38, 51). Meta-analyses demonstrated that the incidence of PVST after colorectal surgery was significantly higher in patients with UC than patients with non-IBD (OR, 4.41; 95% CI, 2.35–8.29; *p* < 0.01), but the incidence of PVST after colorectal surgery was not significantly different between patients with CD and non-IBD (OR, 1.71; 95% CI, 0.25–11.73; *p* = 0.59).

#### Risk Factors of PVST in Patients With IBD After Colorectal Surgery

**Figure 2:**
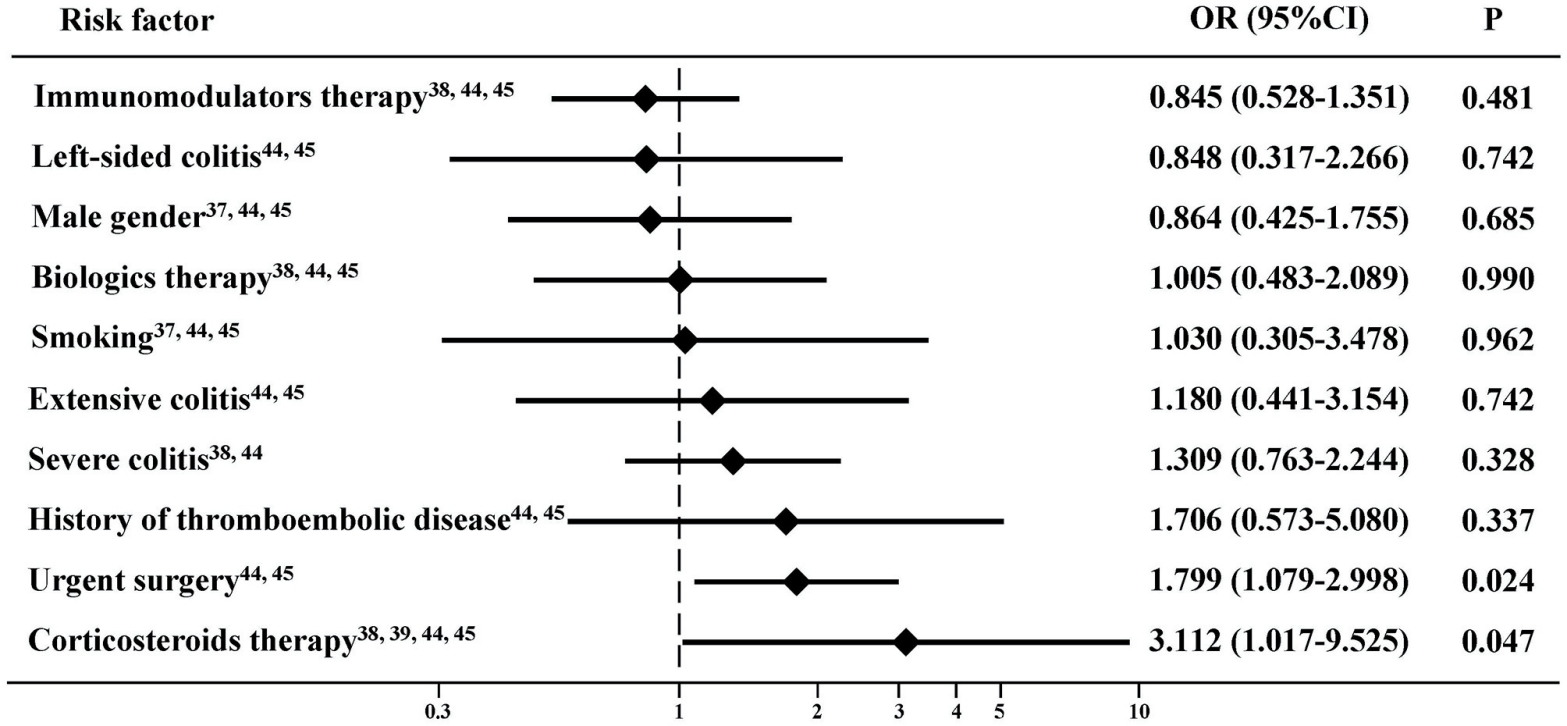
Risk factors of portal venous system thrombosis (PVST) in patients with inflammatory bowel disease (IBD) after colorectal surgery.

A total of six studies demonstrated that age (44), BMI (49), corticosteroids therapy (44), preoperative C-reactive protein (CRP) (45), preoperative albumin (44), surgical approach (49), type of surgery (44), and urgent reoperation (45) were significant risk factors of PVST in patients with IBD after colorectal surgery on the univariate analysis (Supplementary Table 9). Two studies demonstrated that corticosteroid therapy (44), preoperative CRP (45), and type of surgery (44) were significant risk factors of PVST in patients with IBD after colorectal surgery on the multivariate analysis (Supplementary Table 10). Meta-analyses found that corticosteroid therapy (38, 39, 44, 45) and urgent surgery (44, 45) were significantly associated with PVST in patients with IBD after colorectal surgery, but not male gender (37, 44, 45), left-sided colitis (44, 45), extensive colitis (44, 45), severe colitis (38, 44), immunomodulators therapy (38, 44, 45), biologics therapy (38, 44, 45), history of thromboembolic disease (44, 45), or smoking (37, 44, 45) (Figure 2).

## Discussion

To the best of our knowledge, this is the first systematic review with a meta-analysis exploring the epidemiology of PVST in patients with IBD and evaluating its risk factors. Our major findings are as follows: first, among the patients with IBD in whom the history of colorectal surgery was unclear, the prevalence of PVST was 0.99, 1.45, and 0.40% in UC, CD, and unclassified IBD, respectively. Notably, the prevalence of PVST became higher in patients with IBD who underwent imaging examinations. Second, among the patients with IBD who underwent colorectal surgery, the incidence of PVST was 6.95, 2.55, and 3.95% in UC, CD, and unclassified IBD, respectively. Notably, the incidence became higher in patients with IBD who underwent imaging examinations after colorectal surgery. Third, the use of preoperative corticosteroids and urgent surgery are significant risk factors of PVST in patients with IBD who underwent colorectal surgery.

IBD is associated with a hypercoagulable state, which enhances the risk of thrombosis. Indeed, 1.01 to 2.14% of patients with IBD have VTE (56, 57). Several major mechanisms for explaining the association between PVST and IBD are as follows (Supplementary Figure 1). First, ulceration and loss of integrity of the normal mucosal barrier in the bowel may lead to microbial invasion or translocation into the portal venous system, leading to pylephlebitis and increasing the risk of PVST (7). Second, the deficiency of anticoagulants, such as protein S, is related to IBD (58). Protein S deficiency is associated with a high risk of VTE (59) and PVST (60). Third, fibrinogen, which may contribute to the development of PVST (61), is increased in active IBD (62). Fourth, tissue plasminogen activator (t-PA) is released from storage sites in vascular endothelial cells as a result of inflammation in patients with IBD (6). An increase in t-PA level is counteracted by a delayed, but sustained increase in plasminogen activator inhibitor-1 (PAI-1) (63), thereby decreasing fibrinolysis (64). Fifth, homocysteine level is significantly higher in patients with IBD than healthy controls (65). Hyperhomocysteinemia can cause hypercoagulability, by increasing tissue factor and factor V levels, reducing t-PA level, and deactivating protein C (66). Sixth, increased platelet count (67) and decreased mean platelet volume (68) in patients with IBD could increase the thrombotic potential risk. Seventh, the initiation and progression of colitis are mainly caused by neutrophil extracellular traps (NETs), which could induce platelet activation to promote thrombotic tendency (69). Studies concerning NETs in the pathogenesis of PVST are scarce, but evidence on the critical role of NETs in thrombosis is comprehensive (70). NETs are released together with peptidylarginine deiminase type IV into the extracellular milieu, leading to thrombus formation in mesenteric venules in mice (71).

Computed tomography (CT) and magnetic resonance imaging (MRI) play an important role in the diagnosis and assessment of non-malignant PVST (72, 73). However, they are not routinely performed in patients with IBD, which potentially underestimates the actual epidemiology of PVST. This study suggested that the prevalence of PVST and incidence of PVST after colorectal surgery would be increased in patients who underwent imaging examinations. Collectively, CT or MRI may be considered in patients with IBD at a high risk of developing PVST.

Corticosteroids are a major treatment option for IBD (74), but they potentiate the risk of VTE in patients with IBD regardless of colorectal surgery (75). The reasons for this association may include the following: first, corticosteroids could enhance the activity of the PAI-1 gene in cell cultures through a corticosteroid-responsive element with enhancer-like properties (76). Activation of the *PAI-1* gene could increase the PAI-1 level, thereby reducing the t-PA level and impairing the fibrinolytic activity (76). Therefore, corticosteroid-induced alterations in fibrinolysis may contribute to a hypercoagulable state. Second, corticosteroids use may be a surrogate marker for more severe disease status. Usually, mild cases can be treated with derivatives of 5-aminosalicylic acid, but severe cases achieve disease remission by corticosteroids (56). Patients with aggressive disease often suffer from abdominal pain or frequent diarrheal stool per day and need bed rest and relative immobility, which lead to a stronger prothrombotic state (77). Thus, some investigators speculate that an increased risk of VTE may be due to the disease activity, rather than corticosteroid use itself. Contrarily, others consider that patients with IBD treated with corticosteroids are more likely to experience a disease flare, thereby increasing the risk of VTE (78). Higgins et al. showed that corticosteroids themselves increased VTE risk regardless of inflammatory conditions (79). Indeed, there is also an increased risk of VTE in general patients and healthy volunteers receiving corticosteroids in the absence of inflammation (80, 81). In our meta-analysis, the preoperative use of corticosteroids seems to be associated with PVST after colorectal surgery (OR = 3.112; *p* = 0.047). Because the use of corticosteroids is often indispensable in patients with IBD (82), anticoagulation should be considered for the prophylaxis of VTE during the period of corticosteroid use (83). However, based on our meta-analysis, the pooled incidence of PVST in patients with UC after colorectal surgery is not lower in patients receiving antithrombotic drugs. Of course, it should be acknowledged that a direct comparison between anticoagulation vs. no anticoagulation is lacking in such patients. Therefore, further studies should explore the role of anticoagulation for the prevention of PVST in patients with IBD receiving corticosteroids.

A high risk of postoperative thromboembolic complications has been observed in patients with IBD undergoing colorectal surgery (84). Colorectal surgery has been identified as a risk factor of VTE (85). However, it is still unclear whether an increased risk of VTE among patients with IBD is specifically attributed to colectomy, disease severity necessitating colectomy, or their combination. Our meta-analysis also demonstrated that the pooled incidence of PVST was obviously higher in patients with UC who underwent colorectal surgery than those in whom the information regarding colorectal surgery was unclear (6.95 vs. 0.99%), suggesting a higher probability of PVST after colorectal surgery. Thrombosis in the portal venous system is associated with a borderline intrinsically hypercoagulable environment, which may result from direct surgical trauma to the colic veins (42). Additionally, the incidence of PVST after colorectal surgery is higher in patients with UC than patients with CD. This may be explained by the fact that patients with UC undergoing colorectal surgery have a larger inflammatory burden, but patients with CD undergo surgery mainly due to stenotic or fistulizing complications (86). Patients with UC undergoing urgent surgery have an over 5-fold increased odds of VTE, despite postoperative heparin (87). This is because patients usually have a flare of IBD at the time of urgent surgery, leading to a prominent risk of VTE (56). Our meta-analysis also demonstrated that urgent surgery might be a risk factor of PVST. Taken together, thromboprophylaxis in surgical patients with IBD should be adopted according to the specific guidelines (88).

This study has some other limitations. First, the heterogeneity among studies was significant in most meta-analyses. It might be from the enrollment period and follow-up duration. However, the source of heterogeneity cannot be identified by subgroup and meta-regression analyses. Second, the specific type of IBD, severity of IBD, number of patients with IBD who underwent imaging examinations, and history of colorectal surgery for IBD were unclear in some studies. Third, there is a high incidence of PVST after colorectal surgery (89). However, among the included studies, no relevant data can be extracted to compare the proportion of PVST between patients with IBD who underwent and did not undergo colorectal surgery.

In conclusion, there is an increased risk of PVST in patients with IBD. Corticosteroids therapy and urgent colorectal surgery both suggest that more severe IBD seems to increase the risk of PVST in patients with IBD. Imaging examinations should be recommended to improve the detection rate of PVST, especially in high-risk patients. Further large-scale prospective1 studies are necessary to clarify the prediction and prevention of PVST in patients with IBD in the future.

## Data Availability Statement

The original contributions presented in the study are included in the article/Supplementary Material, further inquiries can be directed to the corresponding author/s.

## Author Contributions

HL contributes to the methodology, formal analysis, investigation, data curation, writing—original draft, and writing—review and editing. ZB, FM, and YW contributes to the formal analysis, investigation, data curation, writing—original draft, and writing—review and editing. LL contributes to the formal analysis, investigation, data curation, and writing—review and editing. AS contributes to the writing—review and editing. EY contributes to the writing—review and editing. XG contributes to the investigation, writing—review and editing, and supervision. XQ contributes to the conceptualization, methodology, formal analysis, investigation, data curation, writing—original draft, writing—review and editing, supervision, and project administration. All authors have made an intellectual contribution to the manuscript and approved the submission of the manuscript.

## Conflict of Interest

The authors declare that the research was conducted in the absence of any commercial or financial relationships that could be construed as a potential conflict of interest.

## Publisher's Note

All claims expressed in this article are solely those of the authors and do not necessarily represent those of their affiliated organizations, or those of the publisher, the editors and the reviewers. Any product that may be evaluated in this article, or claim that may be made by its manufacturer, is not guaranteed or endorsed by the publisher.

## Supplementary Material

Supplementary Figure 1 Mechanisms of the association between PVST and IBD.

Supplementary Table 1 Quality of cohort studies. Q1, Representativeness of the exposed cohort; Q2, Selection of the non-exposed cohort; Q3, Ascertainment of exposure; Q4, Demonstration that outcome of interest was not present at start of study; Q5, Comparability of cohorts on the basis of the design or analysis; Q6, Assessment of outcome; Q7, Was follow-up long enough for outcomes to occur; Q8, Adequacy of follow up of cohorts.

Supplementary Table 2 Quality of cross-sectional studies. Q1, Define the source of information (survey, record review); Q2, List inclusion and exclusion criteria for exposed and unexposed subjects (cases and controls) or refer to previous publications; Q3, Indicate time period used for identifying patients; Q4, Indicate whether or not subjects were consecutive if not population-based; Q5, Indicate if evaluators of subjective components of study were masked to other aspects of the status of the participants; Q6, Describe any assessments undertaken for quality assurance purposes (e.g., test/retest of primary outcome measurements); Q7, Explain any patient exclusions from analysis; Q8, Describe how confounding was assessed and/or controlled; Q9, If applicable, explain how missing data were handled in the analysis; Q10, Summarize patient response rates and completeness of data collection; Q11, Clarify what follow-up, if any, was expected and the percentage of patients for which incomplete data or follow-up was obtained. Y, Yes; U, Unclear; N, No.

Supplementary Table 3 Characteristics of included patients with PVST in whom the information regarding colorectal surgery was unclear (*n* = 244). ^a^PVST was not located in one position. PVST, Portal venous system thrombosis; Pts, Patients.

Supplementary Table 4 Results of meta-regression analyses regarding the prevalence of PVST in IBD patients in whom the information regarding colorectal surgery was unclear. PVST, Portal venous system thrombosis; IBD, Inflammatory bowel disease; CD, Crohn's disease.

Supplementary Table 5 Sensitivity analyses in studies where the information regarding colorectal surgery was unclear. CI, Confidence interval; CD, Crohn's disease; IBD, Inflammatory bowel disease.

Supplementary Table 6 Characteristics of included patients with PVST after colorectal surgery (*n* = 789). ^a^PVST was not located in one position. ^b^The hematological abnormalities in the four patients were thrombocytosis, G20210A prothrombin mutation, antithrombin III mutation and anti-phospholipid syndrome, respectively. PVST, Portal venous system thrombosis; Pts, Patients.

Supplementary Table 7 Results of meta-regression analyses regarding the incidence of PVST in patients after colorectal surgery. PVST, Portal venous system thrombosis; UC, Ulcerative colitis; USA, United States of America; IBD: Inflammatory bowel disease.

Supplementary Table 8 Sensitivity analyses in studies where the information regarding colorectal surgery was clear. CI, Confidence interval; UC, Ulcerative colitis; IBD, Inflammatory bowel disease.

Supplementary Table 9 Univariate analysis of risk factors for PVST in IBD patients after colorectal surgery. PVST, Portal venous system thrombosis; IBD, Inflammatory bowel disease; TAC, Total abdominal colectomy; CP, Completion proctectomy; TPC, Total proctocolectomy; IPAA, Ileal pouch-anal anastomosis; RPC, Restorative proctocolectomy; ASA, American Society of Anesthesiologists classification.

Supplementary Table 10 Multivariate analysis of risk factors for PVST in IBD patients after colorectal surgery. PVST, Portal venous system thrombosis; IBD, Inflammatory bowel disease; RPC, Restorative proctocolectomy; IPAA, Ileal pouch-anal anastomosis; CP, Completion proctectomy; TAC, Total abdominal colectomy.
