# Supplementary figures and images for: Epidemiology and Risk Factors of Portal Venous System Thrombosis in Patients With Inflammatory Bowel Disease: A Systematic Review and Meta-Analysis
Source: Front Med (Lausanne). 2022 Jan 17;8:744505. doi: 10.3389/fmed.2021.744505 (PMC8801813; doi:10.3389/fmed.2021.744505)

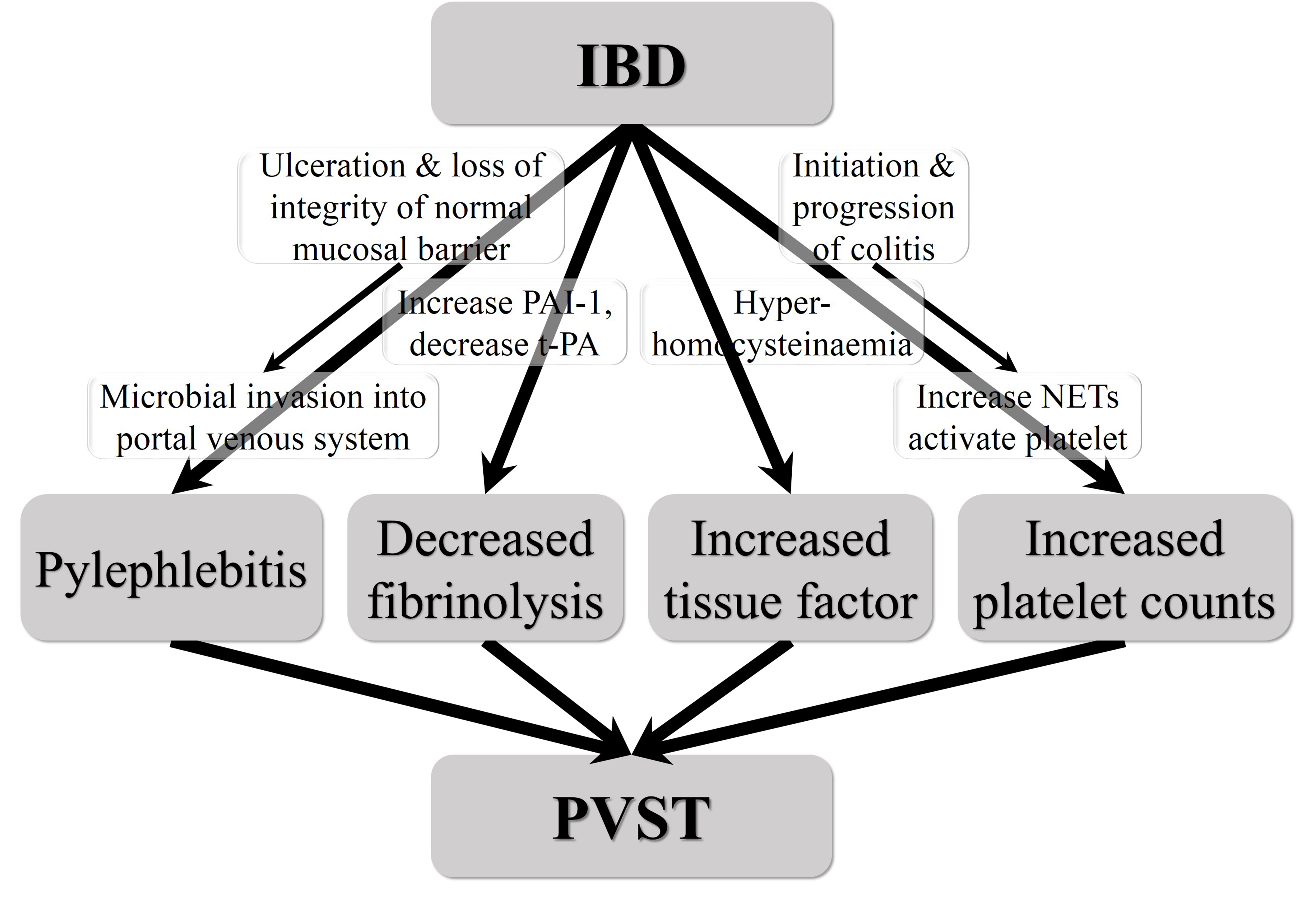

Supplement: Supplementary Figure 1 — Mechanisms of the association between PVST and IBD. [file Image_1.JPEG]
