# Supplementary material for: Epidemiology and Risk Factors of Portal Venous System Thrombosis in Patients With Inflammatory Bowel Disease: A Systematic Review and Meta-Analysis
Source: Front Med (Lausanne). 2022 Jan 17;8:744505. doi: 10.3389/fmed.2021.744505 (PMC8801813; doi:10.3389/fmed.2021.744505)
Supplement: Supplementary Table 1 — Quality of cohort studies. Q1, Representativeness of the exposed cohort; Q2, Selection of the non-exposed cohort; Q3, Ascertainment of exposure; Q4, Demonstration that outcome of interest was not present at start of study; Q5, Comparability of cohorts on the basis of the design or analysis; Q6, Assessment of outcome; Q7, Was follow-up long enough for outcomes to occur; Q8, Adequacy of follow up of cohorts. [file Table_1.docx]

| **Supplementary Table 1. Quality of cohort studies** | | | | | | | | | | | |
| --- | --- | --- | --- | --- | --- | --- | --- | --- | --- | --- | --- |
| **First author (year)** | **Selection** | | | |  | **Comparability** |  | **Outcome** | | | **Total** |
|  | **Q1** | **Q2** | **Q3** | **Q4** |  | **Q5** |  | **Q6** | **Q7** | **Q8** |  |
| Allaix (2014) | / | ☆ | ☆ | ☆ |  | ☆☆ |  | ☆ | ☆ | ☆ | 8 |
| Ball (2007) | / | ☆ | ☆ | ☆ |  | / |  | ☆ | ☆ | ☆ | 6 |
| Bence (2020) | / | ☆ | ☆ | ☆ |  | ☆☆ |  | ☆ | ☆ | ☆ | 8 |
| Bonnivard (2015) | / | ☆ | ☆ | ☆ |  | ☆ |  | ☆ | ☆ | ☆ | 7 |
| Bruining (2008) | / | ☆ | ☆ | ☆ |  | / |  | ☆ | ☆ | ☆ | 6 |
| Feuerstein (2017) | / | ☆ | ☆ | ☆ |  | ☆☆ |  | ☆ | ☆ | ☆ | 8 |
| Fichera (2003) | / | ☆ | ☆ | ☆ |  | / |  | ☆ | ☆ | ☆ | 6 |
| Gonzales (2010) | / | ☆ | ☆ | ☆ |  | / |  | ☆ | ☆ | ☆ | 6 |
| Gu (2016) | / | ☆ | ☆ | ☆ |  | ☆☆ |  | ☆ | ☆ | ☆ | 8 |
| Kayal (2019) | / | ☆ | ☆ | ☆ |  | ☆☆ |  | ☆ | ☆ | ☆ | 8 |
| Kopylov (2012) | / | ☆ | ☆ | ☆ |  | / |  | ☆ | ☆ | ☆ | 6 |
| Mathis (2011) | / | ☆ | ☆ | ☆ |  | ☆☆ |  | ☆ | ☆ | ☆ | 8 |
| Mathis (2013) | / | ☆ | ☆ | ☆ |  | / |  | ☆ | ☆ | ☆ | 6 |
| Medress (2007) | / | ☆ | ☆ | ☆ |  | / |  | ☆ | / | / | 4 |
| Murphy (2013) | / | ☆ | ☆ | ☆ |  | ☆☆ |  | ☆ | / | / | 6 |
| Naik (2011) | / | ☆ | ☆ | ☆ |  | ☆ |  | ☆ | / | / | 5 |
| Papay (2013) | / | ☆ | ☆ | ☆ |  | ☆☆ |  | ☆ | ☆ | ☆ | 8 |
| Ribas Andrade (2016) | / | ☆ | ☆ | ☆ |  | / |  | ☆ | / | / | 4 |
| Robinson (2015) | / | ☆ | ☆ | ☆ |  | ☆☆ |  | ☆ | ☆ | ☆ | 8 |
| Sabban (2017) | / | ☆ | ☆ | ☆ |  | ☆ |  | ☆ | ☆ | ☆ | 7 |
| Soteriadou (2013) | / | ☆ | ☆ | ☆ |  | / |  | ☆ | / | / | 4 |
| Syed  (2021) | ☆ | ☆ | ☆ | ☆ |  | ☆☆ |  | ☆ | ☆ | ☆ | 9 |
| Talbot (1986) | / | ☆ | ☆ | ☆ |  | / |  | ☆ | ☆ | ☆ | 6 |
| Vaidya (2020) | / | ☆ | ☆ | ☆ |  | ☆☆ |  | ☆ | / | / | 6 |
| Vegh (2014) | ☆ | ☆ | ☆ | ☆ |  | ☆☆ |  | ☆ | ☆ | ☆ | 9 |
| Violi (2014) | / | ☆ | ☆ | ☆ |  | ☆☆ |  | ☆ | ☆ | ☆ | 8 |
| Weisshof (2019) | / | ☆ | ☆ | ☆ |  | / |  | ☆ | ☆ | ☆ | 6 |
| Zaghiyan (2012) | / | ☆ | ☆ | ☆ |  | / |  | ☆ | ☆ | ☆ | 6 |
| **Notes:**  **Q1:** Representativeness of the exposed cohort;  **Q2:** Selection of the non-exposed cohort;  **Q3:** Ascertainment of exposure;  **Q4:** Demonstration that outcome of interest was not present at start of study;  **Q5:** Comparability of cohorts on the basis of the design or analysis;  **Q6:** Assessment of outcome;  **Q7:** Was follow-up long enough for outcomes to occur;  **Q8:** Adequacy of follow up of cohorts. | | | | | | | | | | | |
