# Supplementary material for: Epidemiology and Risk Factors of Portal Venous System Thrombosis in Patients With Inflammatory Bowel Disease: A Systematic Review and Meta-Analysis
Source: Front Med (Lausanne). 2022 Jan 17;8:744505. doi: 10.3389/fmed.2021.744505 (PMC8801813; doi:10.3389/fmed.2021.744505)
Supplement: Supplementary Table 2 — Quality of cross-sectional studies. Q1, Define the source of information (survey, record review); Q2, List inclusion and exclusion criteria for exposed and unexposed subjects (cases and controls) or refer to previous publications; Q3, Indicate time period used for identifying patients; Q4, Indicate whether or not subjects were consecutive if not population-based; Q5, Indicate if evaluators of subjective components of study were masked to other aspects of the status of the participants; Q6, Describe any assessments undertaken for quality assurance purposes (e.g., test/retest of primary outcome measurements); Q7, Explain any patient exclusions from analysis; Q8, Describe how confounding was assessed and/or controlled; Q9, If applicable, explain how missing data were handled in the analysis; Q10, Summarize patient response rates and completeness of data collection; Q11, Clarify what follow-up, if any, was expected and the percentage of patients for which incomplete data or follow-up was obtained. Y, Yes; U, Unclear; N, No. [file Table_2.docx]

| **Supplementary Table 2. Quality of cross-sectional studies** | | | | | | | | | | | | |
| --- | --- | --- | --- | --- | --- | --- | --- | --- | --- | --- | --- | --- |
| **First author (year)** | **Q1** | **Q2** | **Q3** | **Q4** | **Q5** | **Q6** | **Q7** | **Q8** | **Q9** | **Q10** | **Q11** | **Total** |
| Ashamalla (2019) | Y | Y | Y | Y | U | Y | N | U | N | N | N | 5 |
| Banerjee (2011) | Y | N | Y | Y | U | Y | N | U | N | N | N | 4 |
| Blonski (2012) | Y | Y | Y | Y | U | Y | Y | U | N | N | N | 6 |
| Campos (2015) | Y | Y | Y | Y | U | Y | N | U | N | N | N | 5 |
| Gutta (2016) | Y | Y | Y | Y | U | Y | Y | U | N | N | N | 6 |
| Heffley (2017) | Y | Y | Y | Y | U | Y | N | U | N | N | N | 5 |
| Leustean (2018) | Y | Y | Y | Y | U | Y | N | U | N | N | N | 5 |
| Mouelhi (2016) | Y | Y | Y | Y | U | Y | N | U | N | N | N | 5 |
| **Notes:**  **Q1:** Define the source of information (survey, record review);  **Q2:** List inclusion and exclusion criteria for exposed and unexposed subjects (cases and controls) or refer to previous publications;  **Q3:** Indicate time period used for identifying patients;  **Q4:** Indicate whether or not subjects were consecutive if not population-based;  **Q5:** Indicate if evaluators of subjective components of study were masked to other aspects of the status of the participants;  **Q6:** Describe any assessments undertaken for quality assurance purposes (e.g., test/retest of primary outcome measurements);  **Q7:** Explain any patient exclusions from analysis;  **Q8:** Describe how confounding was assessed and/or controlled;  **Q9:** If applicable, explain how missing data were handled in the analysis;  **Q10:** Summarize patient response rates and completeness of data collection;  **Q11:** Clarify what follow-up, if any, was expected and the percentage of patients for which incomplete data or follow-up was obtained. **Abbreviations:** Y: Yes; U: Unclear; N: No. | | | | | | | | | | | | |
