# Supplementary material for: Epidemiology and Risk Factors of Portal Venous System Thrombosis in Patients With Inflammatory Bowel Disease: A Systematic Review and Meta-Analysis
Source: Front Med (Lausanne). 2022 Jan 17;8:744505. doi: 10.3389/fmed.2021.744505 (PMC8801813; doi:10.3389/fmed.2021.744505)
Supplement: Supplementary Table 3 — Characteristics of included patients with PVST in whom the information regarding colorectal surgery was unclear (n = 244). aPVST was not located in one position. PVST, Portal venous system thrombosis; Pts, Patients. [file Table_3.docx]

| **Supplementary Table 3. Characteristics of included patients with PVST in whom the information regarding colorectal surgery was unclear (n=244)** | |
| --- | --- |
| **Characteristics** | **No. Pts. (Percentage)** |
| **Gender** | |
| Male/Female/Unclear | 101 (41.39%) / 80 (32.79%) / 63 (25.82%) |
| **Location ^a^** | |
| Main portal vein | 106 (43.44%) |
| Splenic vein | 1 (0.41%) |
| Mesenteric vein and branches | 147 (60.25%) |
| Unclear | 38 (15.57%) |
| **Main clinical presentation** | |
| Abdominal pain | 2 (0.82%) |
| Perianal fistula | 10 (4.10%) |
| Internal fistula | 7 (2.87%) |
| Perianal abscess | 7 (2.87%) |
| Abdominal abscess | 6 (2.46%) |
| Bowel stenosis | 24 (9.84%) |
| Splenomegaly | 2 (0.82%) |
| Unclear | 186 (76.22%) |
| **Hematological abnormalities** | |
| Positive | 38 (15.57%) |
| *Coagulopathy* | 37 (15.16%) |
| *Lupus anti-coagulant* | 1 (0.41%) |
| Negative | 3 (1.23%) |
| Unclear | 203 (83.20%) |
| **Treatment selection** | |
| Anticoagulation/Surgery/Unclear | 123 (50.41%) / 63 (25.82%) / 58 (23.77%) |
| **Outcome** | |
| Alive/Died/Unclear | 11 (4.51%) / 3 (1.23%) / 230 (94.26%) |
| **Notes:** **^a^**: PVST was not located in one position. **Abbreviations:** PVST: Portal venous system thrombosis; Pts: Patients. | |
