# Supplementary material for: Epidemiology and Risk Factors of Portal Venous System Thrombosis in Patients With Inflammatory Bowel Disease: A Systematic Review and Meta-Analysis
Source: Front Med (Lausanne). 2022 Jan 17;8:744505. doi: 10.3389/fmed.2021.744505 (PMC8801813; doi:10.3389/fmed.2021.744505)
Supplement: Supplementary Table 4 — Results of meta-regression analyses regarding the prevalence of PVST in IBD patients in whom the information regarding colorectal surgery was unclear. PVST, Portal venous system thrombosis; IBD, Inflammatory bowel disease; CD, Crohn's disease. [file Table_4.docx]

| **Supplementary Table 4. Results of meta-regression analyses regarding the prevalence of PVST in IBD patients in whom the information regarding colorectal surgery was unclear** | |
| --- | --- |
| **Variables** | **P-value** |
| **CD** | |
| Region (Europe versus North America versus South America versus Asia) | 0.938 |
| Publication year (Before 2015 versus After 2015) | 0.722 |
| Study design (Population-based cohort versus Hospital-based cohort) | 0.659 |
| Sample size (≤400 versus >400) | 0.380 |
| Whether the detailed number of patients undergoing imaging examinations was reported (Yes versus Unclear) | 0.380 |
| Study quality (High versus Moderate) | 0.232 |
| **Unclassified of IBD** | |
| Region (Europe versus North America versus South America versus Africa) | 0.816 |
| Publication year (Before 2015 versus After 2015) | 0.442 |
| Study design (Hospital-based cohort versus Cross-sectional) | 0.505 |
| Sample size (≤400 versus >400) | 0.613 |
| Severity of IBD (Exacerbation versus Unclear) | <0.001 |
| Whether the detailed number of patients undergoing imaging examinations was reported (Yes versus Unclear) | <0.001 |
| Study quality (High versus Moderate) | 0.648 |
| **Abbreviations:** PVST: Portal venous system thrombosis; IBD: Inflammatory bowel disease; CD: Crohn's disease. | |
