# Supplementary material for: Epidemiology and Risk Factors of Portal Venous System Thrombosis in Patients With Inflammatory Bowel Disease: A Systematic Review and Meta-Analysis
Source: Front Med (Lausanne). 2022 Jan 17;8:744505. doi: 10.3389/fmed.2021.744505 (PMC8801813; doi:10.3389/fmed.2021.744505)
Supplement: Supplementary Table 5 — Sensitivity analyses in studies where the information regarding colorectal surgery was unclear. CI, Confidence interval; CD, Crohn's disease; IBD, Inflammatory bowel disease. [file Table_5.docx]

| **Supplementary Table 5. Sensitivity analyses in studies where the information regarding colorectal surgery was unclear** | | | |
| --- | --- | --- | --- |
| **Study omitted** | **Prevalence (95%CI)** | **I^2^** | **P** |
| **CD** | | | |
| Bruining (2008) | 0.0143 (0.0009-0.0277) | 92.90% | <0.0001 |
| Kopylov (2012) | 0.0156 (0.0016-0.0296) | 93.00% | <0.0001 |
| Ribas Andrade (2016) | 0.0179 (0.0023-0.0336) | 93.00% | <0.0001 |
| Soteriadou (2013) | 0.0195 (0.0036-0.0354) | 93.30% | <0.0001 |
| Vegh (2014) | 0.0222 (0.0047-0.0397) | 91.80% | <0.0001 |
| Violi (2014) | 0.0077 (0.0021-0.0133) | 66.90% | 0.0167 |
| **Unclassified IBD** | | | |
| Ashamalla (2019) | 0.0012 (0.0006-0.0018) | 21.80% | 0.2423 |
| Blonski (2012) | 0.0077 (0.0031-0.0122) | 94.40% | <0.0001 |
| Bonnivard (2015) | 0.0040 (0.0013-0.0068) | 94.60% | <0.0001 |
| Campos (2015) | 0.0047 (0.0017-0.0076) | 94.60% | <0.0001 |
| Gutta (2016) | 0.0047 (0.0016-0.0077) | 94.60% | <0.0001 |
| Heffley (2017) | 0.0039 (0.0012-0.0066) | 94.60% | <0.0001 |
| Leustean (2018) | 0.0040 (0.0013-0.0068) | 94.60% | <0.0001 |
| Mouelhi (2016) | 0.0039 (0.0012-0.0066) | 94.60% | <0.0001 |
| Papay (2013) | 0.0046 (0.0015-0.0076) | 94.50% | <0.0001 |
| Sabban (2017) | 0.0040 (0.0013-0.0066) | 94.60% | <0.0001 |
| Talbot (1986) | 0.0072 (0.0029-0.0115) | 94.60% | <0.0001 |
| **Abbreviations:** CI: Confidence interval; CD: Crohn's disease; IBD: Inflammatory bowel disease. | | | |
