# Supplementary material for: Epidemiology and Risk Factors of Portal Venous System Thrombosis in Patients With Inflammatory Bowel Disease: A Systematic Review and Meta-Analysis
Source: Front Med (Lausanne). 2022 Jan 17;8:744505. doi: 10.3389/fmed.2021.744505 (PMC8801813; doi:10.3389/fmed.2021.744505)
Supplement: Supplementary Table 6 — Characteristics of included patients with PVST after colorectal surgery (n = 789). aPVST was not located in one position. bThe hematological abnormalities in the four patients were thrombocytosis, G20210A prothrombin mutation, antithrombin III mutation and anti-phospholipid syndrome, respectively. PVST, Portal venous system thrombosis; Pts, Patients. [file Table_6.docx]

| **Supplementary Table 6. Characteristics of included patients with PVST after colorectal surgery (n=789)** | |
| --- | --- |
| **Characteristics** | **No. Pts. (Percentage)** |
| **Gender** | |
| Male/Female/Unclear | 99 (12.55%) / 60 (7.60%) / 630 (79.85%) |
| **Location ^a^** | |
| Main portal vein | 20 (2.53%) |
| Portal vein branches | 31 (3.93%) |
| Portal vein peripheral | 47 (5.96%) |
| Splenic vein | 3 (0.38%) |
| Mesenteric vein and branches | 38 (4.82%) |
| Unclear | 681 (86.31%) |
| **Main clinical presentation** | |
| Abdominal pain | 46 (5.83%) |
| Nausea | 3 (0.38%) |
| Vomiting | 1 (0.13%) |
| Prolonged ileus | 22 (2.79%) |
| Fever | 7 (0.89%) |
| Malaise | 2 (0.25%) |
| Bleeding from the stoma | 6 (2.76%) |
| Anastomotic stricture | 1 (0.13%) |
| Ileal pouch-anal anastomotic leakage | 3 (0.38%) |
| Abdominopelvic sepsis | 6 (0.76%) |
| Wound infection | 11 (1.39%) |
| Small bowel obstruction | 1 (0.13%) |
| Dehydration/sodium depletion | 8 (1.01%) |
| Abdominal abscess | 1 (0.13%) |
| Unclear | 671 (85.04%) |
| **Interval from surgery to PVST** | |
| ≤30 days/>30 days/Unclear | 45 (5.70%) / 2 (0.25%) / 742 (94.04%) |
| **Hematological abnormalities** | |
| Positive ^b^/Negative/Unclear | 4 (0.51%) / 2 (0.25%) / 783 (99.24%) |
| **Treatment selection** | |
| Anticoagulation/Surgery/Unclear | 83 (10.52%) / 6 (0.76%) / 700 (88.72%) |
| **Outcome** | |
| Alive/Died/Unclear | 29 (3.68%) / 34 (4.31%) / 726 (92.02%) |
| **Notes:**  **^a^**: PVST was not located in one position.  ^b^: The hematological abnormalities in the four patients were thrombocytosis, G20210A prothrombin mutation, antithrombin III mutation and anti-phospholipid syndrome, respectively. **Abbreviations:** PVST: Portal venous system thrombosis; Pts: Patients. | |
