# Supplementary material for: Epidemiology and Risk Factors of Portal Venous System Thrombosis in Patients With Inflammatory Bowel Disease: A Systematic Review and Meta-Analysis
Source: Front Med (Lausanne). 2022 Jan 17;8:744505. doi: 10.3389/fmed.2021.744505 (PMC8801813; doi:10.3389/fmed.2021.744505)
Supplement: Supplementary Table 7 — Results of meta-regression analyses regarding the incidence of PVST in patients after colorectal surgery. PVST, Portal venous system thrombosis; UC, Ulcerative colitis; USA, United States of America; IBD: Inflammatory bowel disease. [file Table_7.docx]

| **Supplementary Table 7. Results of meta-regression analyses regarding the incidence of PVST in patients after colorectal surgery** | |
| --- | --- |
| **Variables** | **P-value** |
| **UC** | |
| Region (USA versus Canada) | 0.660 |
| Publication year (Before 2015 versus After 2015) | 0.190 |
| Study design (Population-based cohort versus Hospital-based cohort) | 0.255 |
| Sample size (≤400 versus >400) | 0.376 |
| Severity of UC (Refractory versus Unclear) | 0.823 |
| Use of antithrombotic drugs (Yes versus Unclear) | 0.868 |
| Whether the indications of imaging examinations for PVST were mentioned (Yes versus Unclear) | 0.292 |
| Whether the detailed number of patients undergoing imaging examinations was reported (Yes versus Unclear) | 0.043 |
| Study quality (High versus Moderate) | 0.078 |
| **Unclassified IBD** | |
| Publication year (Before 2015 versus After 2015) | 0.189 |
| Sample size (≤400 versus >400) | 0.727 |
| Severity of IBD (Refractory versus Unclear) | 0.275 |
| Use of antithrombotic drugs (Yes versus Unclear) | 0.008 |
| Whether the indications of imaging examinations for PVST were mentioned (Yes versus Unclear) | 0.007 |
| Whether the detailed number of patients undergoing imaging examinations was reported (Yes versus Unclear) | 0.010 |
| Study quality (High versus Moderate) | 0.473 |
| **Abbreviations:** PVST: Portal venous system thrombosis; UC: Ulcerative colitis; USA: United States of America; IBD: Inflammatory bowel disease. | |
