# Supplementary material for: Epidemiology and Risk Factors of Portal Venous System Thrombosis in Patients With Inflammatory Bowel Disease: A Systematic Review and Meta-Analysis
Source: Front Med (Lausanne). 2022 Jan 17;8:744505. doi: 10.3389/fmed.2021.744505 (PMC8801813; doi:10.3389/fmed.2021.744505)
Supplement: Supplementary Table 8 — Sensitivity analyses in studies where the information regarding colorectal surgery was clear. CI, Confidence interval; UC, Ulcerative colitis; IBD, Inflammatory bowel disease. [file Table_8.docx]

| **Supplementary Table 8. Sensitivity analyses in studies where the information regarding colorectal surgery was clear** | | | |
| --- | --- | --- | --- |
| **Study omitted** | **Incidence (95%CI)** | **I^2^** | **P** |
| **UC** | | | |
| Allaix (2014) | 0.0745 (0.0342-0.1148) | 93.10% | <0.0001 |
| Ball (2007) | 0.0654 (0.0300-0.1007) | 93.70% | <0.0001 |
| Feuerstein (2017) | 0.0791 (0.0366-0.1215) | 94.10% | <0.0001 |
| Gonzales (2010) | 0.0551 (0.0233-0.0868) | 92.60% | <0.0001 |
| Kayal (2019) | 0.0656 (0.0311-0.1001) | 91.70% | <0.0001 |
| Robinson (2015) | 0.0666 (0.0309-0.1023) | 93.70% | <0.0001 |
| Syed (2021) | 0.0789 (0.0480-0.1099) | 79.00% | <0.0001 |
| Vaidya (2020) | 0.0706 (0.0352-0.1060) | 94.30% | <0.0001 |
| **Unclassified IBD** | | | |
| Bence (2020) | 0.0415 (0.0264-0.0567) | 51.90% | 0.0343 |
| Fichera (2003) | 0.0392 (0.0257-0.0526) | 50.70% | 0.0392 |
| Gu (2016) | 0.0293 (0.0218-0.0369) | 0% | 0.7378 |
| Mathis (2013) | 0.0386 (0.0256-0.0515) | 48.90% | 0.0474 |
| Mathis (2011) | 0.0409 (0.0269-0.0550) | 51.80% | 0.0347 |
| Medress (2007) | 0.0427 (0.0280-0.0574) | 49.70% | 0.0439 |
| Murphy (2013) | 0.0435 (0.0300-0.0570) | 28.10% | 0.1946 |
| Naik (2011) | 0.0384 (0.0251-0.0517) | 48.60% | 0.0489 |
| Weisshof (2019) | 0.0390 (0.0262-0.0518) | 49.50% | 0.0446 |
| Zaghiyan (2012) | 0.0398 (0.0265-0.0530) | 51.80% | 0.0344 |
| **Abbreviations:** CI: Confidence interval; UC: Ulcerative colitis; IBD: Inflammatory bowel disease. | | | |
