# Supplementary material for: Epidemiology and Risk Factors of Portal Venous System Thrombosis in Patients With Inflammatory Bowel Disease: A Systematic Review and Meta-Analysis
Source: Front Med (Lausanne). 2022 Jan 17;8:744505. doi: 10.3389/fmed.2021.744505 (PMC8801813; doi:10.3389/fmed.2021.744505)
Supplement: Supplementary Table 9 — Univariate analysis of risk factors for PVST in IBD patients after colorectal surgery. PVST, Portal venous system thrombosis; IBD, Inflammatory bowel disease; TAC, Total abdominal colectomy; CP, Completion proctectomy; TPC, Total proctocolectomy; IPAA, Ileal pouch-anal anastomosis; RPC, Restorative proctocolectomy; ASA, American Society of Anesthesiologists classification. [file Table_9.docx]

| **Supplementary Table 9. Univariate analysis of risk factors for PVST in IBD patients after colorectal surgery** | | |
| --- | --- | --- |
| **Variables** | **No. studies evaluating the variable in univariate analysis** | **No. studies reporting the variable as a significant risk factor in univariate analysis** |
| **Demographic characteristics** | | |
| *Age* | 4 | 1 |
| *Sex* | 4 | 0 |
| *Body mass index* | 3 | 1 |
| *Race* | 1 | 0 |
| **Past history** | | |
| *Smoking* | 2 | 0 |
| *Oral contraceptives* | 1 | 0 |
| *Previous major abdominal surgery* | 1 | 0 |
| *History of thromboembolic disease* | 2 | 0 |
| **Characteristics of IBD** | | |
| *Duration* | 2 | 0 |
| *Extent* | 2 | 0 |
| *Severity* | 2 | 0 |
| **Preoperative medical therapy** | | |
| *None* | 1 | 0 |
| *Aminosalicylic acid* | 1 | 0 |
| *Corticosteroids* | 4 | 1 |
| *Immunomodulators* | 3 | 0 |
| *Biologics* | 3 | 0 |
| *Anticoagulation* | 3 | 0 |
| **Hematological indices** | | |
| *Preoperative hemoglobin* | 2 | 0 |
| *Preoperative white cell count* | 1 | 0 |
| *Preoperative platelet count* | 2 | 0 |
| *Preoperative C-reaction protein* | 1 | 1 |
| **Liver function** | | |
| *Preoperative albumin* | 2 | 1 |
| *Postoperative liver function* | 1 | 0 |
| **Characteristics of colorectal surgery** | | |
| *Acuity of surgery (Urgent versus Elective)* | 2 | 0 |
| *Surgical approach (Laparoscopic or Open)* | 2 | 1 |
| *Type of surgery (unspecified)* | 4 | 0 |
| *Type of surgery (TAC versus CP)* | 1 | 0 |
| *Type of surgery (CP versus TPC+IPAA)* | 1 | 1 |
| *Type of surgery (RPC versus TAC+IPAA)* | 1 | 1 |
| **Intraoperative information** | | |
| *ASA* | 1 | 0 |
| *Blood transfusion* | 1 | 0 |
| *Tension on the small bowel mesentery* | 1 | 0 |
| *Total operative time* | 1 | 0 |
| **Postoperative complications** | | |
| *Anastomotic leak* | 1 | 0 |
| *Pelvic sepsis* | 1 | 0 |
| *Small bowel obstruction* | 1 | 0 |
| **Urgent reoperation** | 1 | 1 |
| **Total length of hospital stay** | 1 | 0 |
| **Abbreviations:** PVST: Portal venous system thrombosis; IBD: Inflammatory bowel disease; TAC: Total abdominal colectomy; CP: Completion proctectomy; TPC: Total proctocolectomy; IPAA: Ileal pouch-anal anastomosis; RPC: Restorative proctocolectomy; ASA: American Society of Anesthesiologists classification. | | |
