# Supplementary material for: Epidemiology and Risk Factors of Portal Venous System Thrombosis in Patients With Inflammatory Bowel Disease: A Systematic Review and Meta-Analysis
Source: Front Med (Lausanne). 2022 Jan 17;8:744505. doi: 10.3389/fmed.2021.744505 (PMC8801813; doi:10.3389/fmed.2021.744505)
Supplement: Supplementary Table 10 — Multivariate analysis of risk factors for PVST in IBD patients after colorectal surgery. PVST, Portal venous system thrombosis; IBD, Inflammatory bowel disease; RPC, Restorative proctocolectomy; IPAA, Ileal pouch-anal anastomosis; CP, Completion proctectomy; TAC, Total abdominal colectomy. [file Table_10.docx]

| **Supplementary Table 10. Multivariate analysis of risk factors for PVST in IBD patients after colorectal surgery** | | |
| --- | --- | --- |
| **Variables** | **No. studies evaluating the variable in multivariate analysis** | **No. studies reporting the variable as a significant risk factor** |
| **Age** | 1 | 0 |
| **Preoperative C-reaction protein** | 1 | 1 |
| **Preoperative albumin** | 1 | 0 |
| **Corticosteroids** | 1 | 1 |
| **Type of surgery** | | |
| *RPC+IPAA versus CP+IPAA* | 1 | 1 |
| *RPC+IPAA versus TAC* | 1 | 1 |
| *TAC versus CP+IPAA* | 1 | 0 |
| **Abbreviations:** PVST: Portal venous system thrombosis; IBD: Inflammatory bowel disease; RPC: Restorative proctocolectomy; IPAA: Ileal pouch-anal anastomosis; CP: Completion proctectomy; TAC: Total abdominal colectomy. | | |
