# Supplementary material for: Epidemiology and Risk Factors of Portal Venous System Thrombosis in Patients With Inflammatory Bowel Disease: A Systematic Review and Meta-Analysis
Source: Front Med (Lausanne). 2022 Jan 17;8:744505. doi: 10.3389/fmed.2021.744505 (PMC8801813; doi:10.3389/fmed.2021.744505)
Supplement: Supplementary file 12 [file Data_Sheet_1.docx]

| **Items** | **Reported on page** |
| --- | --- |
| **Reporting of background should include** |  |
| Problem definition | 3 |
| Hypothesis statement | 3 |
| Description of study outcome(s) | 3 |
| Type of exposure or intervention used | 3 |
| Type of study designs used | 3 |
| Study population | 3 |
| **Reporting of search strategy should include** |  |
| Qualifications of searchers (eg, librarians and investigators) | Title page |
| Search strategy, including time period included in the synthesis and keywords | 4 |
| Effort to include all available studies, including contact with authors | 4 |
| Databases and registries searched | 4 |
| Search software used, name and version, including special features used (eg, explosion) | 4 |
| Use of hand searching (eg, reference lists of obtained articles) | 4 |
| List of citations located and those excluded, including justification | 4, Figure 1 |
| Method of addressing articles published in languages other than English | N/A |
| Method of handling abstracts and unpublished studies | N/A |
| Description of any contact with authors | Title page |
| **Reporting of methods should include** |  |
| Description of relevance or appropriateness of studies assembled for assessing the hypothesis to be tested | 5, 6 |
| Rationale for the selection and coding of data (eg, sound clinical principles or convenience) | 5, 6 |
| Documentation of how data were classified and coded (eg, multiple raters, blinding, and interrater reliability) | 5, 6 |
| Assessment of confounding (eg, comparability of cases and controls in studies where appropriate) | 5, 6 |
| Assessment of study quality, including blinding of quality assessors; stratification or regression on possible predictors of study results | 5, 6 |
| Assessment of heterogeneity | 5, 6 |
| Description of statistical methods (eg, complete description of fixed or random effects models, justification of whether the chosen models account for predictors of study results, dose-response models, or cumulative meta-analysis) in sufficient detail to be replicated | 5, 6 |
| Provision of appropriate tables and graphics | Tables 1-4, Figure 1, 2 and Supplementary materials |
| **Reporting of results should include** |  |
| Graphic summarizing individual study estimates and overall estimate | N/A |
| Table giving descriptive information for each study included | Table 1, 4 |
| Results of sensitivity testing (eg, subgroup analysis) | Table 2, 4, and Supplementary materials |
| Indication of statistical uncertainty of findings | Table 2, 4 |
| **Reporting of discussion should include** |  |
| Quantitative assessment of bias (eg, publication bias) | Table 2, 4 |
| Justification for exclusion (eg, exclusion of non–English-language citations) | Figure 1 |
| Assessment of quality of included studies | Supplementary materials |
| **Reporting of conclusions should include** |  |
| Consideration of alternative explanations for observed results | 12-15 |
| Generalization of the conclusions (ie, appropriate for the data presented and within the domain of the literature review) | 12 |
| Guidelines for future research | 14, 15 |
| Disclosure of funding source | Title page |

**MOOSE Statement—Checklist of items that should be included in reports of *Meta-analysis Of Observational Studies in Epidemiology (MOOSE)***

**Abbreviations:** N/A: Not applicable.
